# Patient Satisfaction and Clinical Effects of Platelet-Rich Plasma on Pattern Hair Loss in Male and Female Patients

**DOI:** 10.7759/cureus.28801

**Published:** 2022-09-05

**Authors:** Samuel P Hetz, Jennifer Martin, Hanno Pototschnig

**Affiliations:** 1 Cosmetic Medicine, Concept Medical, Ottawa, CAN; 2 Regenerative Medicine, Beauty & Lips, Munich, DEU

**Keywords:** trichology, platelet-rich plasma, hair loss, female pattern hair loss, dermatology, autologous conditioned plasma, alopecia, androgenetic alopecia, aesthetics, acp

## Abstract

Introduction

Hair loss is a widespread condition in both genders. Over the past decade, platelet-rich plasma (PRP) has become a common treatment for hair loss. Our goal was to analyze patient satisfaction and the clinical effects of PRP on male and female pattern hair loss.

Methods

Over a period of 12 months, we treated a total of 56 patients for androgenetic alopecia with PRP. All of these patients were included in this study; 15 cc of whole blood was spun in an ACP double syringe (Arthrex Inc., Naples, Forida) for five minutes. The yielded 5 to 6 cc of PRP were then injected into the scalp. Forty-nine patients were treated with a series of three injections at monthly intervals, three patients with a series of four injections, two patients with a series of five injections, one patient was treated with a series of seven injections, and one patient with a series of eight injections. Follow-ups were conducted one month after the last treatment. A self-drawn questionnaire was used to assess the satisfaction and clinical results from the patient's and the clinician's perspectives.

Results

The average age was 41 years (20-68 years). Fifty-seven percent of all patients were male and 43% female. In total, the patients were satisfied with the treatment results. The average score was 7.29 on a scale from 0 to 10. The clinician's rating was similar (6.46). Moreover, with an average score of 8 on a scale from 0 to 10, it is very likely that the patients will recommend PRP treatments to friends. The probability of occurrence of clinical effects among the entire study population was reported as follows: improvement in hair density (patient‘s rating: 64%; clinician's rating: 46%), thickness (38%; 45%), quality (46%; 54%), sheen/lustre (27%; 21%), new hair growth (57%; 68%), less hair loss (48%; 20%), other positive effects (5%, 2%), no effects (4%; 4%), negative effects (0%; 0%).

Conclusion

Our study revealed encouraging results for the treatment of male and female pattern hair loss with PRP. The autologous treatment was rated with high satisfaction scores and can be considered a safe and effective treatment modality.

## Introduction

Hair loss is a widespread condition in the western world. Particular roles are played by androgenetic alopecia, with a prevalence of up to 80% in caucasian males and 50% in females [1]. In women, androgenetic alopecia may be regarded as being of pathological origin, whereas androgenetic alopecia occurs so commonly in males that it is regarded as a normal characteristic of aging [1, 2]. Common approaches for treating pattern hair loss include oral 5α-reductase inhibitors (i.e., finasteride) and thus the reduction of testosterone into dihydrotestosterone, and the topical application of minoxidil, which shortens the resting phase (telogen phase) of the hair cycle, with the result that the growth phase (anagen phase) is reached faster and the growth of new hair stimulated. Both treatments are frequently associated with poor patient compliance, as they need to be performed on a daily basis, and side effects (e.g., impotence, depression) can occur with finasteride in particular [3]. Several years ago, low-level laser therapy (LLLT) was proposed as an alternative treatment option. Among various mechanisms, the main mechanism is hypothesized to be the stimulation of epidermal stem cells in the hair follicle bulge and the shifting of the follicles into the anagen phase. In general, LLLT has demonstrated a low incidence of adverse effects. Still, LLLT is not as widespread as the two above-mentioned treatments [4]. Over the past decade, platelet-rich plasma (PRP) has become a common treatment for hair loss. The PRP's growth factors and cytokines are supposed to initiate wound healing. Research demonstrated antiapoptotic effects on dermal papilla cells and that fibroblast growth factor-7 and B-catenin prolong the anagen phase of the hair cycle. [5]. From a clinical point of view, evidence demonstrating positive effects from PRP treatments is growing [6]. Still, PRP treatments are usually not reimbursed by healthcare insurance, and patients must pay the costs of treatment. Hence, not only objective treatment success but also patient satisfaction is particularly important. Therefore, we investigated the satisfaction with PRP treatments as the primary endpoint.

## Materials and methods

**Figure 1:**
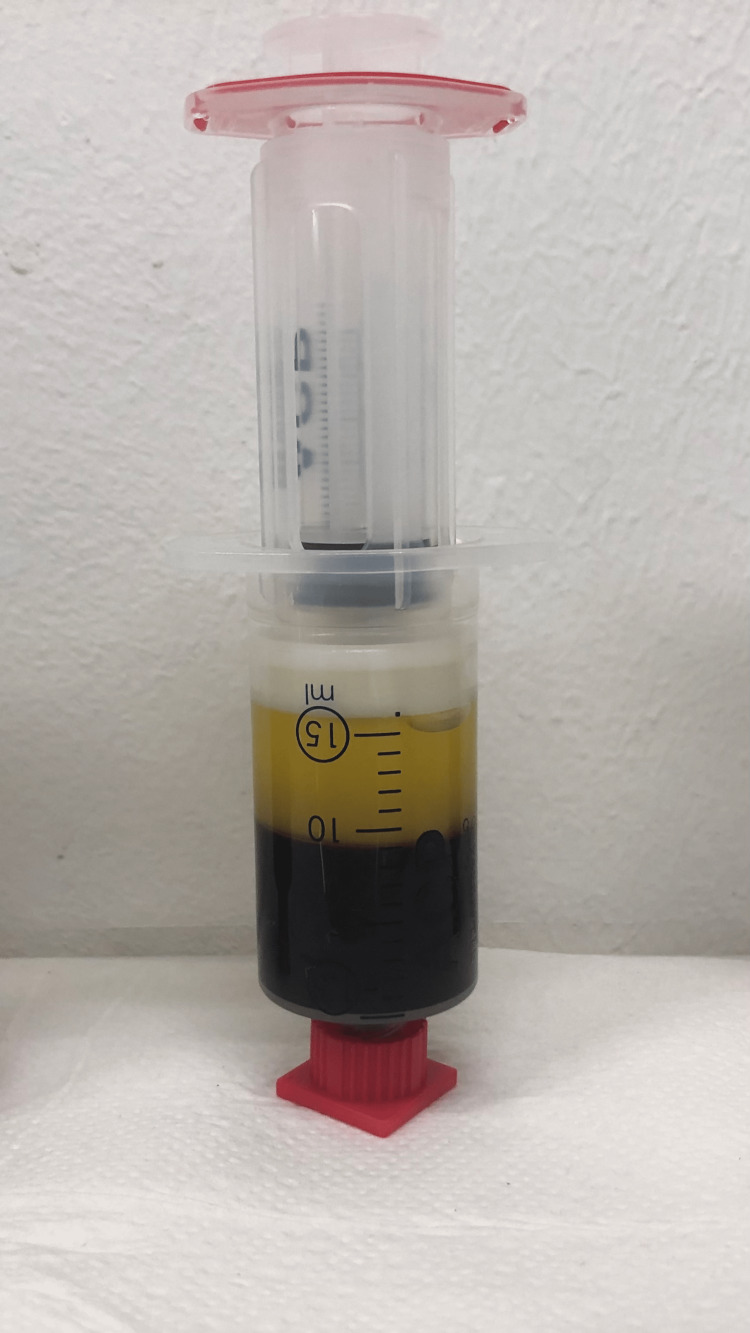
Preparation of platelet-rich plasma

Over a period of 12 months, we treated a total of 56 patients for male and female pattern hair loss with PRP. All of the patients were included in this retrospective study. Specific inclusion/exclusion criteria did not exist. The principles outlined in the Declaration of Helsinki were followed; consent was obtained from all participants. All patients were treated according to their individual needs. As part of the daily routine, we collected satisfaction and clinical data. All data was anonymized and evaluated retrospectively for this study. A serial treatment consisting of a minimum of three sessions at monthly intervals was suggested to the patients. No supplements were given to the patients. Fifteen cc of whole blood was aspired from the patient via a butterfly needle into an ACP double syringe (Arthrex Inc., Naples, Florida) at each session. Then, the syringe was centrifuged in a HORIZON 24-AH centrifuge (Drucker Diagnostics LLC., Philipsburg, Pennsylvania) for five minutes at 1500 rpm (350 G) horizontally. The PRP settled in the upper third of the syringe (approx. 5 to 6 cc) and was aspired into the inner syringe (Figure 1). The inner syringe was unscrewed, and after disinfection, the PRP was injected in serial puncture technique 1 cm apart into the scalp at a depth of approx. 3 mm. Platelets were not activated exogenously (e.g., through the addition of calcium gluconate) in our protocol. According to literature, PRP prepared with the ACP double syringe is poor in leucocytes and erythrocytes and has a platelet concentration of approx. 2.5 times over baseline [7].

Satisfaction scores and clinical results were evaluated one month after the last treatment. The primary endpoint of the study was patient satisfaction. Patients were asked to rate their satisfaction with the PRP treatment on a numeric rating scale (How satisfied are you with your PRP treatment results? 0 = not satisfied at all; 10 = extremely satisfied). Secondary endpoints had been the recommendation rate (How likely are you to recommend PRP to a friend or family member? 0 = not likely at all; 10 = extremely likely), the self-evaluation of clinical effects (Has PRP improved any of the following? Circle all that apply: density, thickness, quality, sheen/luster, new hair growth, less hair loss, other, none of above), the treating clinician’s evaluation (Has PRP improved any of the following? Circle all that apply: density, thickness, quality, sheen/luster, new hair growth, less hair loss, other, none of above), and clinician’s ratings (How satisfied are you with your patient‘s PRP treatment results? 0 = not satisfied at all; 10 = extremely satisfied), as well as the collection of negative effects/serious adverse events. The satisfaction and recommendation scores were analyzed separately for male and female pattern hair loss. Welch’s t-test was performed to analyze the differences for statistical significance (p<0.05).

## Results

**Figure 2:**
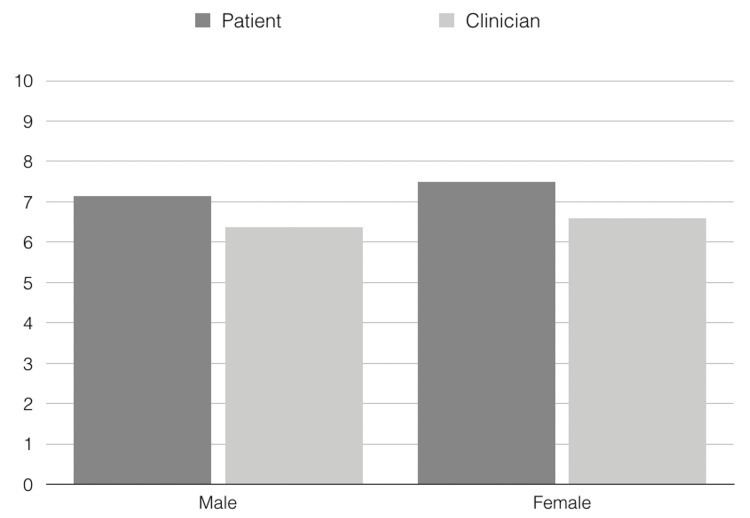
Patient's and clinician's satisfaction after platelet-rich plasma treatment of male and female pattern hair loss

**Figure 3:**
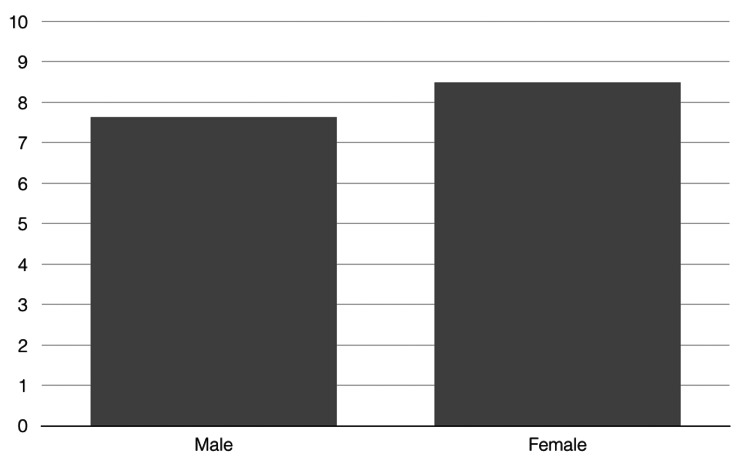
Likeliness of patient's recommendation of platelet-rich plasma treatments for male and female pattern hair loss

The average age of all 56 patients was 41 years (20-68 years). Fifty-seven percent of all patients were male, and 43% were female. Forty-nine patients were treated with a series of three injection sessions, three patients with a series of four sessions, two patients with a series of five sessions, one patient was treated with a series of seven sessions, and one patient with a series of eight sessions. Adverse events did not occur; all patients completed the study. In total, the patients were satisfied with the treatment results. The average score was 7.29 on a scale from 0 to 10 (males: 7.13, females: 7.5). There was no statistically significant difference between male and female pattern hair loss (p=0.9003). The clinician's rating was similar (total group: 6.46, males: 6.38, females: 6.58.) (Figure 2). Also, for this rating, the differences between the genders were not statistically significant (p=0.9452). Moreover, the difference between patients' ratings was not statistically significant (p=0.78). With an average score of eight (males: 7.63, females: 8.5) on a scale from 0 to 10, it is very likely that the patients will recommend PRP treatments to friends (Figure 3). For this rating, statistically significant differences from a gender perspective could not be detected (p=0.7123).

The probability of occurrence of clinical effects among the entire study population is presented in Table 1.

**Table 1:** Probability of occurrence of clinical effects

Clinical effect	Patient's assessment	Clinician's assessment
Improved hair density	64%	46%
Improved thickness	38%	45%
Improved quality	46%	54%
Improved sheen/luster	27%	21%
New hair growth	57%	68%
Less hair loss	48%	20%
Other positive effects	5%	2%
No effects	4%	4%
Negative effects	0%	0%

## Discussion

**Figure 4:**
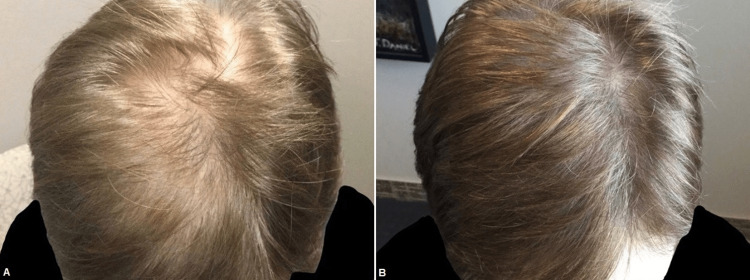
Male patient before (A) and one month after three platelet-rich plasma treatments (B)

**Figure 5:**
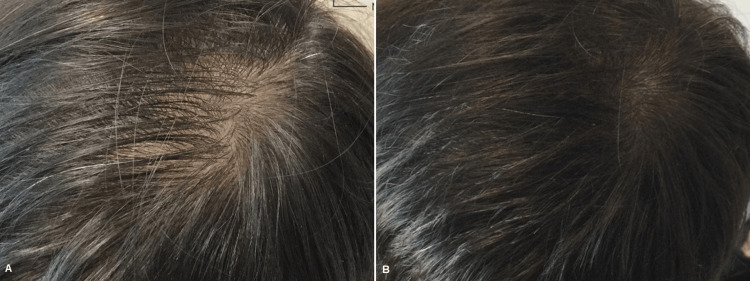
Male patient before (A) and one month after three platelet-rich plasma treatments (B)

**Figure 6:**
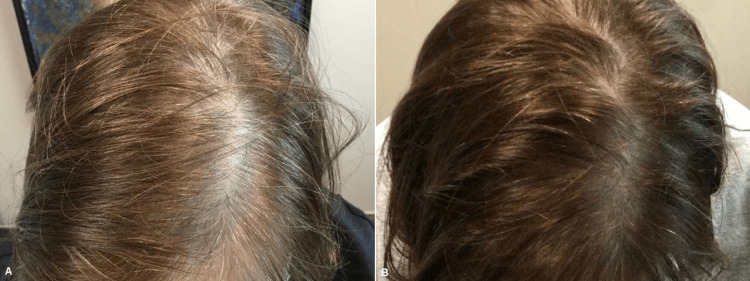
Female patient before (A) and one month after three platelet-rich plasma treatments (B)

**Figure 7:**
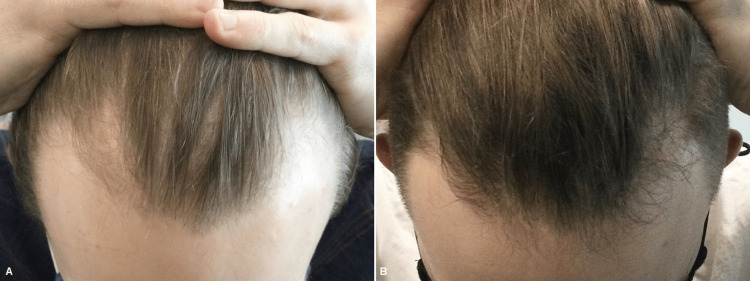
Female patient before (A) and one month after three platelet-rich plasma treatments (B)

**Figure 8:**
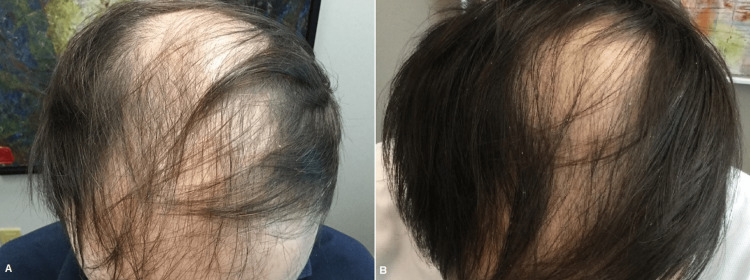
Male patient before (A) and one month after three platelet-rich plasma treatments (B)

According to literature, PRP is a low-risk intervention to treat male and female pattern hair loss (androgenetic alopecia) and is associated with objective improvements in outcomes such as good patient satisfaction [6,8,9]. Evans et al. recently reviewed 30 studies, including 687 patients on PRP for the treatment of androgenetic alopecia. Twenty-nine studies reported beneficial results, and 24 studies reached statistical significance on a measured outcome. Ten RCTs were included. The meta-analyses showed that PRP treatment increases hair density and hair thickness. The authors concluded that PRP is an autologous treatment that lacks serious adverse effects and effectively improves hair density and hair thickness in men and women with androgenetic alopecia [8]. Concerning the treatment of female pattern hair loss with PRP, Zhou et al. stated that PRP showed excellent efficiency through hair density evaluation. In their systematic review and meta-analyses, a total of 42 studies of 1,569 cases, including 776 female participants covering 16 randomized controlled trials and 26 observational trials, were included. In comparison to the control groups with an odds ratio (OR) of 1.61, 95% CI 0.52-2.70, and in comparison to baseline with OR 1.11, 95% CI 0.86-1.37, PRP demonstrated effective outcomes concerning hair density in treating female pattern hair loss [9]. Revealing a high patient satisfaction and positive clinical ratings, our study is in line with current literature. Our average patient satisfaction score was 7.29 on a scale from 0 to 10. In four other studies, a scale from 0 to 10 had been used as well. Gkini et al. reported a mean patient satisfaction score of 7.1 out of 10, Tawfik and Osman, Khatu et al., and Betsi et al. reported a score of seven out of 10 [10-13]. Moreover, Hausauer and Jones reported a patient satisfaction mean score of 2.3 (on a 0-3 scale) [14]. In contrast to the existing studies, we assessed the clinician's satisfaction the first time and could demonstrate that patient's and clinician's satisfaction are very similar. Still, the unblinded and uncontrolled setting represents a limitation of our study. Concerning the clinical signs, improved hair density was the most frequent sign from the patient's perspective (64%) and new hair growth (68%) from the clinician's perspective (Figures 4-8).

Certainly, our clinical (self) evaluation is not as precise as trichoscopic imaging, but the fact that in only 4% of all our cases no improvements could be detected from both patient's and clinician's perspective is still remarkable. Although pattern hair loss is predominantly a male problem, almost half of our patients were female, which suggests a particularly high-level willingness to undergo a treatment modality that is privately paid. Finally, as reported already in literature, our data suggests that treatment efficacy is similar for male and female pattern hair loss.

In our study, we investigated a serial mono-therapy with PRP. Recently protocols combining PRP with other treatment modalities were published. PRP combined with minoxidil 5% revealed superior results concerning hair density, mean hair count, anagen and telogen percentages, and mean anagen/telogen ratio, compared to the combination of PRP and finasteride 1 mg (p< .05: PRP plus minoxidil vs. PRP plus finasteride) in a randomized placebo-controlled, double-blind, half-head study [15]. According to Shah et al., microneedling with PRP plus topical minoxidil 5% has been significantly more effective than topical minoxidil 5% alone [16]. Furthermore, Gowda et al. reported statistically superior results of PRP plus minoxidil vs. minoxidil alone and vs. a dermaroller plus minoxidil [17]. Another comparative study reported good response in the minoxidil 5% side in 41%, moderate response in 20%, and poor response in 39%, whereas for the PRP plus minoxidil 5% side good response has been reported in 59%, moderate response in 16%, and poor response in 25% of the patients [18]. Moreover, Vaaruni et al. reported excellent improvement for 60% of their patients in the Minoxidil plus PRP group vs. 33.33% in the minoxidil mono-therapy group [19]. Gupta et al. published a systematic review with network meta-analyses comparing various non-surgical treatment modalities. PRP had the highest surface under the cumulative ranking curve (SUCRA) value (96.07%) in the network for male androgenetic alopecia, while placebo (or sham) had the lowest (1.73%) (Table 2).

**Table 2:** Surface under the cumulative ranking curve (SUCRA) values in the network for male androgenetic alopecia PRP - platelet-rich plasma, LLLT - low-level laser therapy, SUCRA - surface under the cumulative ranking curve

Treatment modality	SUCRA values
PRP	96%
LLLT	80%
Dutasteride 0.5 mg	70%
Finasteride 1 mg	58%
Minoxidil 5%	50%
Minoxidil 2%	28%
Bimatoprost	16%
Placebo/sham	2%

PRP was significantly more efficacious than dutasteride, finasteride, minoxidil, bimatoprost, and placebo, and tendentially more effective than LLLT. In the network for female androgenetic alopecia PRP, dutasteride and finasteride were not included. LLLT had the highest SUCRA value (97.05%) out of the three included treatment modalities, while placebo had the lowest (0.01%) (Table 3).

**Table 3:** Surface under the cumulative ranking curve (SUCRA) values in the network for female androgenetic alopecia LLLT - low-level laser therapy, SUCRA - surface under the cumulative ranking curve

Treatment modality	SUCRA values
LLLT	97.05%
Minoxidil 5%	51.68%
Minoxidil 2%	51.29%
Placebo/sham	0.01%

Compared to placebo, all these three treatments were significantly more effective, but there was no statistically significant difference between the three treatments [20].

## Conclusions

Our study revealed encouraging results for the treatment of pattern hair loss with PRP for both men and women. Although the prevalence of androgenetic alopecia is higher in males, almost half of our patients were females, which suggests a particularly high-level willingness to undergo this serial treatment modality that is privately paid. The autologous treatment was rated with high satisfaction scores without the occurrence of adverse events and can be considered a safe and effective treatment modality. As patient satisfaction scores are subjective to some extent, we added clinician satisfaction scores and found out that they were consistent with the scores from the patients. Our study confirms the positive patient satisfaction scores and the high safety profile of PRP injections reported in literature. Therefore, we consider PRP a valuable treatment option for male and female pattern hair loss. Nevertheless, future randomized controlled studies investigating both hair count and patient satisfaction would be desirable.
